# Hypercapnia outcome in COVID-19 acute respiratory distress syndrome patients on mechanical ventilator: A retrospective observational cohort

**DOI:** 10.2478/jccm-2025-0004

**Published:** 2025-01-31

**Authors:** Sarwat Rasheed, Sidra Javed, Thanyat Rasheed, Shaiza Farman, Elisha Shalim

**Affiliations:** Sindh Infectious Disease Hospital and Research Centre, Karachi, Pakistan; Dow University of Health Sciences, Karachi, Pakistan; Patel Hospital, Karachi, Pakistan

**Keywords:** hypercapnia, ARDS, mortality, weighted regression

## Abstract

**Introduction:**

Acute respiratory distress syndrome (ARDS) is characterized by progressive lung inflammation which leads to increased dead space that can cause hypercapnia and can increase the risk of patient morbidity and mortality. In an attempt to improve ARDS patient outcomes provision of protective lung ventilation has been shown to improve patient mortality but increases the incidence of hypercapnia. Therefore, the role of carbon dioxide in ARDS remains contradicted by conflicted evidence. This study aims to examine this conflicting relationship between hyper-capnia and mortality in mechanically ventilated COVID-19 ARDS patients.

**Methods:**

We conducted a retrospective cohort study. The data was collected from the medical records of the patients admitted with COVID-19 ARDS in Sindh Infectious Disease Hospital & Research Centre (SIDH & RC) from August 2020 to August 2022 and who received mechanical ventilation for more than 48 hours. The patients were grouped into severe and no severe hypercapnia groups based on their arterial blood carbon dioxide levels (PaCO2). To understand the effect of hypercapnia on mortality we performed multivariable logistic regression, and inverse probability-weighted regression to adjust for time-varying confounders.

**Results:**

We included 288 patients to detect at least 3% of the effect on mortality. Our analysis revealed an association of severe hypercapnia with severe lung injury, low PaO2/FiO2, high dead space, and poor compliance. In univariate analysis severe hypercapnia showed higher mortality: OR=3.50, 95% CI (1.46–8.43). However, after, adjusting for disease severity hypercapnia is not found to be associated with mortality: OR=1.08, 95% CI (0.32–3.64). The sensitive analysis with weighted regression also shows no significant effect on mortality: OR=1.04, 95% CI (0.95–1.14).

**Conclusion:**

This study showed that hypercapnia is not associated with mortality in COVID-19 ARDS patients.

## Introduction

### Background

Acute respiratory distress syndrome (ARDS) is a syndrome of inflammation and increased permeability of capillaries triggered by pulmonary and non-pulmonary insult with clinical, radiological, and physiological abnormality [1]. Apart from primarily lung injury, several patients with ARDS also develop multiple organ failure due to the inflammatory and immune-mediated mechanisms triggered by ARDS. Furthermore, the literature also revealed that multiple organ failure in ARDS patients with COVID-19 infection is also caused by direct viral invasion [2]. Protective lung ventilation strategies, that is reduction in tidal volume and limiting airway pressures, reduce lung inflammation [3] and therefore, decrease mortality and organ failure in ARDS patients during mechanical ventilation and improve outcomes [4]. However, the change in ventilation practice worldwide to low tidal volume in ARDS increased the incidence of hypercapnia [5]. Experimental studies in vitro animal models and in vivo show a beneficial effect of hypercapnia by decreasing inflammatory response [6]. On the other hand, the ARDS in COVID-19 patients has predominant and severe endothelial involvement, which causes thrombotic microangiopathy, micro thrombosis neo angiogenic of pulmonary capillaries leading to worsening of dead space [7] and refractory hypercapnia [8]. Recent studies in vitro and in vivo animal studies also showed hypercapnia has adverse outcomes impairing pulmonary epithelium healing, skeletal muscle protein anabolism, diaphragmatic dysfunction, impaired neutrophil phagocytosis, and decreased immune response [9].

A secondary analysis of three prospective non-interventional cohorts performed by Nicolas Nin et al. [10] showed that the patients with moderate to severe ARDS with severe hypercapnia which is PaCO2 equal to or above 50 mm Hg during the first 48 hours of mechanical ventilation have higher mortality rates as compared to no severe hypercapnia patients with PaCO2 less than 50 mmHg. However, a PRoVENTCOVID observational study [11] which was done to study ventilator practice during the pandemic of COVID-19 showed no mortality difference between hypercapnia and normocapnia patients. Due to the conflicting evidence in the literature on the effects of hypercapnia on patient mortality, we design this study. The purpose of this study is to explore the impact of persistent hypercapnia in mechanical ventilated ARDS due to COVID-19.

### Study Aim

To explore the effect of persistent hypercapnia on mortality during protective lung ventilation strategy in mechanically ventilated COVID-19 ARDS patients.

## Methodology

### Study design and setting

To investigate the impact of hypercapnia in COVID-19 ARDS patients, we devised a retrospective observational cohort. The medical records of patients admitted to the Intensive care unit (ICU) of Sindh Infectious Disease Hospital & Research Centre (SIDH & RC) from August 2020 to August 2022 were explored for this study. We used Strengthening the reporting of observational studies in epidemiology study (Strobe) guidelines to present this study.

### Participant inclusion and exclusion criteria

For this study, we included patients with confirmed COVID-19 by real-time reverse transcription polymerase chain reaction (RT-PCR) assay for SARS-CoV-2 and on mechanical ventilator due to ARDS.

The participants were selected through random sampling from the patient medical records, admitted from August 2020 to August 2022.

We excluded the following patients;

1. Age less than 18 years and more than 80 years.
2. Pregnancy.
3. Patient with DNR (don’t resuscitate) code.
4. Patient on a mechanical ventilator for less than 48-h.
5. The patient was shifted to another hospital.
6. Patients with missing arterial blood gases.

### Pre-specified variables

The exposure in this study is severe hypercapnia, which is the PaCO2 in blood gas above or equal to 50 mmHg.

The primary outcome of this study is Hospital and ICU mortality. Secondary outcomes will include duration of mechanical ventilation, need for renal replacement therapy, venous thromboembolism, hemodynamic instability (norepinephrine of more than 0.05 mic/kg/min, vasopressin, adrenaline), hospital-acquired infections, and length of hospital stay.

For this study, we adopted the diagnostic criteria for ARDS as proposed by Micheal et al. for the new global definition of ARDS [12] which includes:

1. Acute onset or new worsening respiratory symptom in the presence of predisposing risk factor for ARDS.
2. Bilateral opacity on chest radiology not explained by effusion, atelectasis, or nodules/masses)
3. Pulmonary edema cannot be explained by cardiac insufficiency or fluid overload.
4. Impair oxygenation (PaO2/FiO2<300 mmHg).

The potential covariates and confounders that were measured and adjusted include;

1. Baseline information of patients like age, sex, medication, and comorbid like hypertension, diabetes mellitus, chronic kidney disease, malignancy, liver cirrhosis, obstructive lung disease, restricted lung disease.
2. Simplified acute physiological score II (SAPS II) to assess disease severity at baseline.
3. Ventilator settings were noted at three fixed points of time in a day; 08 hours, then 16 hours, and then 01 hours, and their mean was calculated. The ventilator settings assessed were tidal volume ml/kg, minute ventilation L/min, positive end-expiratory pressure PEEP cm H2O, driving pressure (plateau pressure-PEEP), lung compliance, and corrected minute ventilation (as a marker of dead space).
4. Treatment received including prone positioning ventilation, use of neuromuscular paralysis, use of noninvasive ventilator before intubation, steroid, Remdesivir, and Tocilizumab.
5. Hemodynamic parameters like blood pressure and heart rate were also recorded at three fixed points of time in a day; 08 hours, then 16 hours, and then 01 hours, and then the means of those readings were used.

### Data sources/measurements

The medical records of patients admitted to the ICU and who had received invasive ventilation due to COVID-19 ARDS were explored for this study. The patients were divided into two groups, severe hypercapnia and no severe hypercapnia based on their PaCO2 levels. The patients with PaCO2 equal to or above 50 mm Hg were grouped as severe hypercapnia while patients with PaCO2 less than 50 mmHg were included in no severe hypercapnia group. We considered an initial five days of mechanical ventilation after 24-h of intubation. Therefore, the first 24 hours of mechanical ventilation were taken as day 0 then from that, we took a further 5 days. Patients having persistent PaCO2 in blood gas equal to or above 50 mmHg for more than two consecutive days were included in the severe hypercapnia group and patients have PaCO2 of 50 mmHg or more on day 5 were included in the severe hypercapnia group if it persisted on day 6 and day 7.

In our ICU, the patient usually has 3-4 arterial blood gas (ABG) performed each day; therefore, we selected the ABG with worse PaCO2.

For comparing baseline characteristics of severe hypercapnia with no severe hypercapnia group we considered:

– ABG before mechanical ventilation
– ABG with mechanical ventilation setting, and hemodynamic parameter 24 hours after mechanical ventilation.

We addressed selection bias through well-defined inclusion and exclusion criteria and ensured an adequate sample. Information bias was minimized by standardization of the data, and prespecified the variable and their source.

1. Laboratory parameters and medication reviewed from the Health Management Information System of DUHS (HMIS)
2. Ventilator setting and lung mechanics from respiratory therapist flow sheet,
3. Hemodynamic parameter from ICU flow sheet,
4. Baseline characteristics, and primary and secondary outcomes recorded from patients’ record files.

To ensure confidentiality and anonymity personal data like name, contact number, ethnicity, and medical record number that can unveil participant identification is not recorded. We used the code number for each medical record generated by the data department as per hospital policy. All data collected and securely stored in line with Dow University of Health Science (DUHS) policy.

### Study size

We calculated the study size to assess the hypercapnia effect on mortality using G power software. We applied two-independent t-test sampling for binary outcomes. Considering a two-sided alpha significance level of 0.05 with at least 80% power, we needed 288 patients to detect the effect of at least 3% in mortality.

### Statistical methods

The data was collected, stored, and analyzed using software of Microsoft Excel, and R Studio in this study.

We expressed a continuous variable median with an interquartile range and the categorical variable was presented as percentile. For comparing severe hypercapnia and no severe hypercapnia patients we used the Mann-Whitney test for the continuous variable while the Fisher Exact test was used for the categorical variable.

The study aims to understand the effect of hypercapnia in arterial blood gasses in COVID ARDS patients on the odds of hospital survival. To estimate the independent effect of hypercapnia on hospital mortality we performed univariate analysis, regressed mortality as the dependent variable to independent variables including baseline characteristics of patients including age, gender, predicted body weight, disease severity at baseline (SAPS II score, PaO2/FiO2 ratio before intubation), comorbid (Charlson comorbidity index), treatment during ICU stay, PaO2/FiO2 ratio, ventilator parameters, and lung mechanics after 24-hours of mechanical ventilation. We also included other factors affecting mortality accruing in the majority of the population including hemodynamic instability, and hospital-acquired infection. The variables showing significant effects in the univariate analysis were included in the multivariable model. Furthermore, for the explanatory multivariate logistic regression model, we added all potential confounding factors as suggested by existing literature and scientific knowledge. We followed the general statistical assumption of linear logit transformation for continuous variables, free of collinearity in covariates Based on the highest area under the receiver operating curve, the final model was concluded.

Ventilator parameters (including tidal volume, respiratory rate, PEEP), and respiratory mechanics (including driving pressure, and plateau pressure) are time-dependent co-variable that could affect both outcome and exposure. Secondly, time-dependent exposure (severe hypercapnia) depends on disease severity at baseline and also on evolution in disease severity.

We conducted a sensitivity analysis to adjust for biased estimates in the presence of time-varying exposure and time-varying covariates. Robin et.al described the marginal structural model using inverse probability of treatment weighted (IPTW) estimation (13) to adjust for these biases in estimation. Therefore, we performed a pooled logistic regression model and estimated propensity score to predict probabilities of severe hypercapnia for each patient. We included the following variables for propensity score:

1. PaO2/FiO2, compliance, dead space, ventilator parameter, and respiratory mechanics at the onset of hypercapnia
2. PaO2/FiO2, SAPS II, and PaCO2 before mechanical ventilation as baseline disease severity
3. PaO2/FiO2, compliance, dead space ventilator parameter, respiratory mechanics at day 1 of mechanical ventilation. We used this propensity score to calculate IPTW which estimates the exposure effect in the entire population. To estimate the effect of hypercapnia on mortality IPTW was added in generalized weighted regression.

## Results

**Fig. 1.**
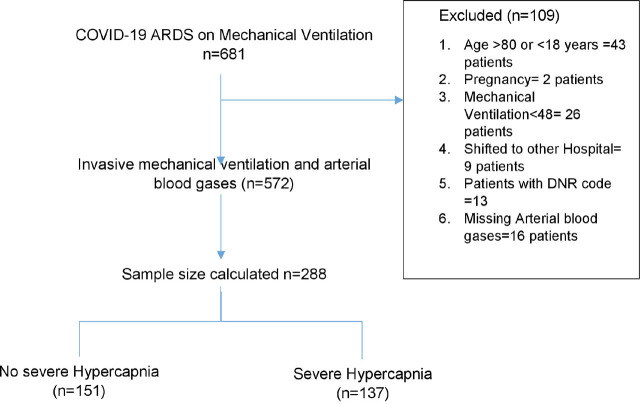
Study Profile

We include 288 patients by random sampling from the medical records of patients admitted to the ICU of SIDH & RC from August 2020 to August 2022 to assess at least a 3% effect of hypercapnia on mortality in COVID-19 ARDS patients on mechanical ventilator. From 288 patients 137 (47.56%) of the patient developed severe hypercapnia. We had 219 (23%) missing data for predicted body weight which was adjusted statistically by multiple imputations. We used the flow chart Figure 1 to demonstrate number of patients excluded.

**Fig. 2.**
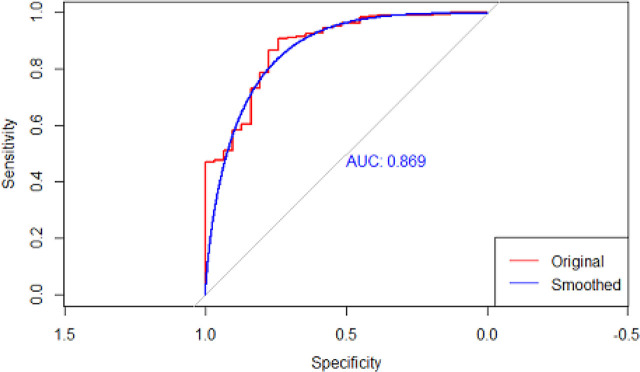
ROC curve for weighted regression

Compared to no severe hypercapnia, severe hypercapnia patients have lower PaO2/FiO2 and higher dead space. Both groups of patients ventilated with similar tidal volume/kg. However, severe hypercapnia patients have higher driving pressure and respiratory rate as shown in Table 1. We also noticed an increased risk of hospital-acquired infection in the severe hypercapnia group. Severe hypercapnia also had prolonged hospital stays but the length of mechanical ventilation was the same in both groups. No difference in other secondary outcomes between the two groups was noticed, as shown in Table 2.

**Table 1.** Clinical characteristics of patients with severe hypercapnia and no severe hypercapnia

	**No severe hypercapnia (n=151)**	**Severe hypercapnia (n=137)**	**p-value**
Age (years)	60 [50.5–69.0]	61 [53, 65]	0.90
Male sex (n (%))	85 (56)	84(61)	0.45
Predicted body weight (kg)	67 [62–72]	64 [58–70]	0.01
Charlson comorbidity index (points)	2 [1–3]	2 [2–3]	0.99
Simplified Acute Physiology Score II (points)	38 [32–42]	38 [33–42]	0.97

**Co-morbid**			
Hypertension (n (%))	86 (57.0)	87 (63.5)	0.31
Asthma (n (%))	9 (6.0	5 (3.6)	0.52
Chronic obstructive lung disease (n (%))	4 (2.6)	7 (5.1)	0.43
Restrictive lung disease (n (%))	1(0.7)	0	1.00
Diabetes mellitus (n (%))	75 (49.7)	66 (48.2)	0.89

**Treatment during ICU stay**			
Prone positioning (n (%))	65(43)	8(58.4)	0.01
Remdesivir (n (%))	114(75)	108 (78)	0.59
Tocilizumab (n (%))	50(33)	56(40)	0.21
Neuromuscular block (n (%))	138 (93.9)	137 (97.2)	0.28
Steroid (n (%))	141 (95.9)	138 (97.9)	0.53
NIV use (n (%))	117(77)	125(91)	0.002
Recruitment (n (%))	11 (7.3)	11 (8)	0.98

**Arterial blood gases (a day before intubation)**			
pH	7.3 (7.3–7.4)	7.4 (7.3_7.4)	0.01
PaCO2 (mmHg)	36 [31–41]	39.9 [35–48]	0.0001
PaO2 (mmHg)	69.1 [56.1–88.0]	63.5 [55–76]	0.03
PaO2/FiO2 (ratio)	79 [63–124]	70 [57–86]	0.000
Lactate	1.8(1.2–2.5)	2(1.2–1.4)	0.73

**Arterial blood gases (after 24 hours of mechanical ventilation)**			
pH	7.3(7.3_7.4)	7.3(7.2–7.4)	0.00
PaCO2 (mmHg)	40(35–45)	54(48–60)	0.00
PaO2 (mmHg)	80.2 [68.9, 103]	73 [65, 86.2]	0.002
Lactate	1.8(1.2–2.5)	2(1.3–2.2)	0.98
PaO2/FiO2 (ratio)	122[ 88–182]	93[ 73–123]	0.001
Tidal volume(ml/kg)	6.1(5.9–6.5)	6(5.8–5.6)	0.08
Tidal volume(ml)	400 [400, 440]	400 [350, 420]	0.000
Set respiratory rate	28 [25–30]	30 [28–35]	0.0001
Minute ventilation	11.2 [ 9.6–12.6]	12 [10–12]	0.07
Applied peep	10.5 (8–12)	10 (8–10)	0.45
Plateau pressure	29 [28, 30]	30 [28, 30]	0.003
Driving pressure	18 [16, 20]	19 [16, 21]	0.03
Static compliance	22 [18–27]	23 [19, 25.0]	0.41
Corrected minute ventilation	11 [ 9.2, 13.4]	16[13, 18.3]	0.00
MAP	92(85–100)	92(85–102)	0.63
Heart rate	100(90–110)	101(90–114)	0.10

**Mortality**	127(84)	130(94)	0.04

The value of the continuous variable is presented as the median with interquartile range (IQR) in parenthesis, and the categorical variable is presented as the percentile in parenthesis

**Table 2.** Secondary outcome in severe hypercapnia and no severe hypercapnia

	**Severe hypercapnia**	**No severe hypercapnia**	**p-value**
Hemodynamic instability	125(82)	115(83)	0.91
Arrhythmia	31 (21)	25 (17.7)	0.79
Acute coronary syndrome	40 (27.2)	32 (22.7)	0.63
Impair liver enzyme	11 (7.2)	8 (5.0)	0.79
Emphysema	15 (9)	23 (16.3)	0.22
Renal replacement therapy	48(31)	29(28)	0.05
Pneumothorax	8 (5)	11 (8)	0.48
Deep vein thrombosis	2 (1.3)	1 (1.4)	1.00
Hospital Acquired Infection	99 (65)	107 (75.9)	0.03
Pulmonary embolism	3(2)	5 (2)	0.13
Ventilator duration	7 [ 4, 10]	6 [ 4, 10]	0.19
Hospital length of stay	11 [ 8, 15]	14 [ 9, 21]	0.04
Ventilator duration in survivals(days)	11(6–21)	6.3(4.5–5.75)	0.065
Hospital length of stay in survivals(days)	34(29–33)	17(14–11)	0.005

The value of the continuous variable is presented as the median with interquartile range (IQR) in parenthesis, and the categorical variable is presented as the percentile in parenthesis

In our study severe hypercapnia when analyzed in univariate analysis show increased mortality with an odds ratio of 3.50 with a 95% confidence interval of 1.46–8.43 and a p-value of 0.005 as shown in Table 3.

**Table 3.** Univariate Regression Analysis

	**OR (95%CI)**	**p-value**
Severe hypercapnia	3.5096 (1.4605, 8.4333)	0.005
Age	1.05 (1.02, 1.09)	0.003
Gender, male	0.84 (0.40, 1.80)	0.6
Predicted body weight	0.96 (0.92, 1.01)	0.11
SAPSII	1.11 (1.06, 1.17)	<0.001
Charlson Comorbidity Index	1.14 (1.06, 1.91)	0.022
Non-invasive ventilation before intubation	2.29 (0.94, 5.20)	0.056
Prone positioning	0.50 (0.22, 1.07)	0.082
Use of steroid	7.47 (1.76, 29.9)	0.004
Use of Tociluzumab	0.79 (0.37, 1.71)	0.5
Use of Remdisivir	0.79 (0.28, 1.90)	0.6
Use of neuromuscular block	8.57 (2.58, 27.8)	<0.001
PaO2/FiO2 ratio before mechanical ventilation	1.00 (0.99, 1.00)	0.2

**After 24-h of ventilation**		
PaO2/FiO2 ratio	0.99 (0.99, 1.00)	0.009
Tidal volume, ml	1.00 (0.99, 1.00)	0.3
Tidal volume, ml/kg	1.18 (0.70, 2.10)	0.5
Minute ventilation	1.19 (1.01, 1.41)	0.042
Respiratory rate	1.09 (1.02, 1.17)	0.011
Driving pressure	0.94 (0.86, 1.03)	0.2
Compliance	1.02 (0.96, 1.08)	0.6
Plateau pressure	1.01 (0.90, 1.14)	0.8
Dead space	1.25 (1.12, 1.41)	<0.001
Applied peep	1.13 (1.00, 1.29)	0.054
Duration of hospital stay	0.94 (0.91, 0.98)	<0.001
Duration of mechanical ventilation	1.02 (0.94, 1.11)	0.7
Acidosis	2.25 (1.06, 4.88)	0.036
Hospital-acquired infections	1.93 (0.88, 4.12)	0.092
Hemodynamic instability	6.36 (2.87, 14.2)	<0.001

To assess the effect of hypercapnia independent of other causes of mortality and confounders that might affect exposure and outcome, a multivariable logistic regression model for adjusted mortality was used. This includes baseline characteristics of patients including age, gender, predicted body weight, disease severity at baseline (SAPS score, PaO2/FiO2 before intubation), comorbid (Charlson comorbidity index), ventilator parameter, and lung mechanics. When adjusting for confounders, as shown in Table 4, severe hypercapnia did not show a significant effect on mortality with an odds ratio of 1.08 with a 95% confidence level of 0.32 – 3.64 with a p-value of 0.89.

**Table 4.** Multivariable Analysis (ROC=0.88)

	**OR**	**95% CI**	**p-value**
Severe hypercapnia	1.08	0.28–3.48	0,993
SAPS II	1.1347	1.05–1.22	0.001
Prone positioning	0.4574	0.15–1.38	0.165
Hospital-acquired infections	1.4012	0.52–3.75	0.503
Hemodynamic instability	7.2308	2.49–20.9	0.000
Dead space	1.2778	1.01–1.60	0.033
Minute ventilation	1.3399	0.94–1.89	0.100
Plateau Pressure	0.9100	0.76–1.07	0.272
Predicted body weight	0.9182	0.85–0.98	0.012
PaO2/FiO2 ratio	0.9922	0.98–1.00	0.125
Charlson Comorbidity Index	0.8413	0.53–1.31	0.451

The sensitive analysis with weighted regression also showed no significant effect on mortality with an odd ratio of 1.04 with a 95% confidence interval of 0.95–1.14 and a p-value of 0.357.

## Discussion

This retrospective study explored the effects of severe hypercapnia on mortality in COVID-19 ARDS during mechanical ventilation. We compared patients with severe hypercapnia with PaCO2 more than or equal to 50 mmHg and no severe hypercapnia. Our study results revealed that hypercapnia by itself does not affect mortality when adjusted for disease severity using logistic regression and IPTW. Kergenow et al also showed in their secondary analysis of the ARDS network study that hypercapnia has no effect on mortality after adjustment for disease severity when ventilated with a tidal volume of 6 ml/kg, hypercapnia exerts no effect on mortality [14].

We found that patients with severe hypercapnia had severe lung disease, manifesting as a low PaO2/FiO2 ratio, high dead space, and high driving pressure, as also demonstrated by other researchers in their studies [15,12]. Our study showed that patients in severe hypercapnia group despite receiving ventilation with similar tidal volume in comparison to no severe hypercapnia group demonstrate higher driving pressure. In the severe hypercapnia group due to severe lung disease despite having tidal volume similar to no severe hypercapnia group there is an increase in driving pressure which increases the stress and strain on lungs thus limiting protective lung ventilation. Furthermore, to achieve protective lung ventilation in the severe hypercapnia group increase the respiratory rate to avoid hypercapnia this can further intensify the stress and strain on the lung. Therefore, to ensure protective lung ventilation extracorporeal life support organization (ESLO) guidelines recommend using extracorporeal membrane oxygenation (ECMO) in hypercapnia with acidosis which does not respond to protective mechanical ventilation [16]. Therefore, we need to consider hypercapnia as a marker of disease severity and view it in the context of underlying cause and disease severity. Anissa et al in their post hoc analysis of the PROvent-COVID-19 ARDS also demonstrated that hypercapnia does not affect mortality [11]. Another, randomized trial on carbon dioxide (CO2) removal in ARDS with Extracorporeal CO2 removal (ECCO2R) showed no effect on mortality despite achieving a 20% reduction in carbon dioxide level from baseline [17].

Moreover, another single-center case series in COVID-19 patients used directed hypercapnia to wean from venovenous extracorporeal membrane oxygenation (VV ECMO) revealed that inducing compensatory respiratory acidosis by decreasing sweep gas flow can create metabolic support for early decannulation of ECMO and that compensated hypercapnia is well tolerated [18]. Recently, another single-center study [19] in cardiac arrest patients showed that patients with hypercapnia have improved cardiac performance, and mixed venous oxygen saturation with no effect on right ventricular function and pulmonary vasculature However, another study conducted by Nin N et.al in non-COVID ARDS demonstrated higher mortality in hypercapnia after adjusting for disease severity [10]. This contraindication in grey literature can be because certain phenotypes of ARDS may respond differently resulting in different outcomes as Liu X, argued in their retrospective analysis of data to identify phenotype clusters in ARDS associated with clinical outcome and treatment response [20]. Further, studies can be done to unveil the impact of different phenotypes of ARDS in which hypercapnia may cause adverse outcomes.

Furthermore, there was no difference in the length of mechanical ventilation between the two groups in our study, the severe hypercapnia group patient’s hospital stay was prolonged as compared to the no severe hypercapnia group patients. The potential reason may be due to the effect of hypercapnia on muscle weakness leading to the prolonged need for rehabilitation. Studies on animal models [21,22] demonstrated that hypercapnia can depress anabolism of skeleton muscle via several mechanisms leading to skeleton muscle weakness. Prolonged hospital stay in patients with severe hypercapnia in our study can be explained by available literature. Herridge et al demonstrated that patients surviving ARDS show a reduction in exercise capacity years after discharge [23]. However, we need further studies to evaluate the direct effect of hypercapnia in ARDS patients on muscle strength.

This study showed an increased incidence of hospital-acquired infection in severe hypercapnia group patients as compared to no severe hypercapnia group patients which can be due to the underlying severe lung disease in this group however, we cannot delineate this from the direct impact of hypercapnia on immune response as Gate et.al showed in their study that hypercapnia impairs lung neutrophil function which can increase mortality in murine pseudomonas pneumonia [24].

Moreover, the present study showed high mortality, 88%, in COVID-19 ARDS patients who received mechanical ventilation which is much higher in comparison to studies conducted in other countries [25,11]. This may be due to delays in hospitalization in our country because of a lack of awareness, scarcity of resources, insufficient number of mechanical ventilators, and shortage of trained ICU physicians [26]. Moreover, the baseline median PaO2/FiO2 of both study groups in our study was less than 80 upon initial presentation while it was above 100 in studies conducted in other countries [11], which can also be due to the lack of awareness to seek early medical attention in our general population [27]. Therefore, this study reports the effect of CO2 on ARDS patients with severe disease.

Our study showed that hypercapnia by itself is not associated with mortality when adjusted for disease severity. We used the marginal structural model to adjust for time-varying confounders that affect outcome and exposure including mechanical ventilation parameters, lung mechanics, and disease severity at the onset of hypercapnia and baseline disease severity. This study does not explore the effects of hypercapnia on pulmonary vasculature, right ventricle, and hemodynamic instability. Since it is an observation study, we also did not evaluate the direct causal effect of hypercapnia on mortality. Although this study gives clinical ground for future Randomized clinical trials on interventions to remove CO2 in patients with severe disease. Despite using the structural model for casual interference in longitudinal data unmeasured confounders cannot be adjusted. Furthermore, the dead space in the lungs was calculated from corrected minute ventilation instead of calculated from capnography due to a lack of resources. Similarly, the static lung compliance was calculated from the formula which is less reliable than using esophagus pressure monitoring and pressure/volume curve. This study was conducted in a single center but to decrease type II error we calculated sample size alpha significance, power, and effect size. However, results may not reflect the outcomes of hypercapnia in COVID-19 ARDS patients on mechanical ventilators in other contexts.

Since it was an observational study, it cannot assess the direct causal interference of CO2 on mortality. Studies on the impact of CO2 along with other severity indexes including PaO2/FiO2 ratio, poor compliance, and high dead space in ARDS mechanically ventilated patients and the use of cell-directed therapy like mesenchymal stem cell to dampen the inflammatory response, can play a vital role to improve the outcome in these patients [28]. Furthermore, randomized trials on ECCO2R for CO2 removal including patients with poor compliance and high dead space can also further assess the overall benefit of ECCO2R in ARDS.

## Conclusion

The hypercapnia is not found to be associated directly with patient mortality. However, hypercapnia reflects disease severity and therefore, can be used as a marker for disease severity along with the other signs of disease severity and underlying cause of disease.
